# Preventive Effects of *Ilex Cornuta* Aqueous Extract on High-Fat Diet-Induced Fatty Liver of Mice

**DOI:** 10.1155/2022/7183471

**Published:** 2022-04-07

**Authors:** Meifang Liu, Haoxin Jia, Yang He, Yu Huan, Zhe Kong, Nuo Xu, Xiangju Cao, YueTong Duan, Zhaoliang Li, Luyuan Yang, Wen-Ping Wei, Lifang Wang, Li Li

**Affiliations:** School of Pharmacy, Jining Medical University, No. 669, Xueyuan Road, Donggang District, Rizhao, Shandong 276826, China

## Abstract

**Objective:**

To investigate the preventive effects of *Ilex cornuta* aqueous extract (ICAE) on high-fat diet (HFD)-induced fatty liver of mice and its mechanisms.

**Materials and Methods:**

Twenty-six male KM (Kunming) mice were divided into 3 groups, including the control group (*n* = 9), fed with normal diet; HFD group (*n* = 9), fed with HFD; ICAE + HFD group (*n* = 8), fed with HFD and administered with ICAE (3 g·kg^−1^·d^−1^) at the same time for 10 weeks. Body weight, liver weight, intra-abdominal and subcutaneous fat weight, serum triglyceride (TG), total cholesterol (TC), and blood glucose were determined to evaluate the preventive effects of ICAE on obesity. The average 24 h food consumption of the mice was monitored for 5 times in the 9th week of the experiment to investigate the effects of ICAE on food intake. Serum alanine transaminase (ALT) and aspartate aminotransferase (AST) were assayed to observe the influences of HFD and ICAE on liver function. HE staining was adopted to observe the influence of ICAE on the morphology of adipose tissue and liver tissue. Hepatic TG and TC content assay and oil red O staining were used to evaluate the influences of ICAE on HFD-induced fatty liver, and the protein expression of peroxisome proliferator-activated receptors *γ* (PPAR*γ*) and adipose differentiation-related protein (ADRP) in liver were examined by immunoblotting.

**Results:**

ICAE treatment significantly reduced the increase of body weight, intra-abdominal, and subcutaneous fat and liver weight induced by HFD (*P* < 0.001), but has no influence on food intake; ICAE treatment attenuated the elevation of serum TG, TC, and glucose, as well as serum ALT and AST (*P* < 0.01, *P* < 0.05, *P* < 0.001) and dramatically decreased the content of TG in liver (*P* < 0.01), but has no influence on hepatic TC content. HE staining and oil red O staining showed that ICAE significantly reduced HFD-induced white adipocyte hypertrophy and significantly inhibited lipid accumulation in liver. Immunoblotting showed that the protein levels of PPAR*γ* and ADRP were significantly increased by HFD induction, which can be dramatically reduced by ICAE treatment (*P* < 0.05, *P* < 0.0001).

**Conclusion:**

ICAE has preventive effects on HFD-induced obesity and fatty liver in mice, exerted beneficial effects upon HFD-induced hepatic injury. The preventive effects of ICAE on fatty liver are concerned with the downregulation of PPAR*γ* and ADRP protein expression in liver.

## 1. Introduction

Nonalcoholic fatty liver disease (NAFLD), which affects both adults and children, is characterized by excess fat accumulation in the liver in the absence of significant alcohol use and is the leading cause of chronic liver disease with a worldwide prevalence of approximately 25% [1, 2]. However, there is no effective treatment for NAFLD other than weight loss through dietary change and exercise so far [3–5]. Simple hepatic steatosis, if not controlled, can gradually develop into steatohepatitis and further develop into liver fibrosis, cirrhosis, and even liver cancer [1]. Therefore, it is urgent to find safe and efficient antiNAFLD drugs. In recent years, traditional Chinese herbs have attracted increasing attention for the treatment of NAFLD due to their unique advantages such as multi-targets and multi-channel mechanisms of action [6, 7].

Chinese herb *Ilex cornuta* is the dry leaves of *I. cornuta Lindl*. *ex Paxt*, also known as “hornthorn tea” and “Gonglao leaf.” It is recorded in the Chinese pharmacopoeia, “*Ilex cornuta*, bitter in taste and cool in nature, has the functions of clearing heat, nourishing Yin, calming the liver, tonifying the kidney and dispelling rheumatism.” *I. cornuta* was traditionally used for the treatment of tuberculosis, weakness of the waist and knees, arthrodynia, rheumatic arthralgia, headache, dizziness, and cardiovascular diseases [8]. Pharmacological studies have revealed that *I. cornuta* has multiple functions, such as antioxidation, anti-inflammation, myocardial protection [9–12], antiobesity, lowering blood lipids, and reducing liver weight [13, 14]. However, whether *I. cornuta* can ameliorate the pathogenesis of fatty liver has not yet been reported.

Accumulating evidence supported that nuclear transcription factors such as peroxisome proliferator-activated receptors *γ* (PPAR*γ*) [15–17] and lipid droplet-associated protein adipose differentiation-related protein (ADRP) [18–20] play important roles in the development of fatty liver. It is reported that ADRP gene transcription is regulated by PPARs in human hepatocytes, and both of them are upregulated in high-fat diet (HFD)-induced fatty liver [21, 22]. Our previous study had proved that ICAE downregulated the protein level of PPAR*γ* and inhibited adipocyte differentiation in vitro [14]. In addition, Feng et al. reported that some of the new monomeric compounds isolated from leaves of *I. cornuta* have the activity of inhibiting the transcription of PPAR*γ* gene [23]. Therefore, we proposed that the antifatty liver effects of ICAE might be associated with the modulation of PPAR*γ* and ADRP expressions.

In the present study, we made aqueous extract from the leaves of *I. cornuta* (ICAE), elucidated the effects of ICAE on fatty liver in an HFD-induced mouse model, and further investigated the influences of ICAE on the protein expression of PPAR*γ* and ADRP to explore the underlying protective mechanisms.

## 2. Materials and Methods

### 2.1. Experimental Animals, Grouping, and Procedure

All experimental procedures complied with the recommendations of ARRIVE (Animal Research: Reporting of In Vivo Experiments) guidelines. The Institutional Animal Care and Use Committee of Jining Medical University approved the procedures. About 40 clean grade male Kunming mice aged 4–5 weeks with body weight of 18–22 g were purchased from Jinan Pengyue Experimental Animal Breeding Co., Ltd. (license no. SCXK(LU)20140007). After feeding with normal diet for 1 week, twenty-six mice with similar body weight (22–26 g) were selected and divided into three groups, including the control group (basal diet + normal saline, *n* = 9), HFD group (HFD + normal saline, *n* = 9), and ICAE treatment group (HFD + ICAE, *n* = 8). Basal diet (no. 88001223) was purchased from Shenyang Maohua Biotechnology Co., Ltd. (license no. SCXK(LIAO)2017-0001), and HFD was homemade by adding lard, egg yolk, and cholesterol to basal diet. ICAE 100 mg·kg^−1^·d^−1^ (equivalent to the crude drug of 3 g·kg^−1^·d^−1^) [13, 14, 24] was administered to mice for 10 weeks through oral gavage. All animals were maintained under 12-hour light/dark conditions, with free access to food and water.

The effect of ICAE on food intake was observed in the 9th week of the experiment for 5 consecutive days. Daily food intake per cage was weighed to calculate the average daily food intake per mouse. In the 10th week, animals were fasted on the night of the last administration, weighed, and sacrificed on the next morning. Blood samples were taken from the orbital vein and serum was isolated for the subsequent biochemical assays. After being sacrificed by cervical dislocation, the epididymal fat, perirenal fat, inguinal subcutaneous fat, and the liver of mice were immediately removed and accurately weighed. Small pieces of epididymal adipose tissue and liver were removed and fixed in 4% paraformaldehyde for subsequent histological examination. The remaining tissues were cut into small pieces, placed in 1.5 mL Eppendorf tubes, and stored in a refrigerator at −80°C. The frozen tissue samples were used for the determination of hepatic TC and TG content, as well as protein expression. Hepatic TG and TC levels were measured with the commercial kit and protein expression was detected by immunoblotting.

### 2.2. Main Reagents

ICAE dry powder and high-fat feed were homemade in the laboratory [14]; cholesterol was purchased from Macklin Inc. (Macklin, Shanghai, China). Oil red O was purchased from Sigma (USA). The commercial kits for serum glucose, triglyceride (TG), total cholesterol (TC), alanine aminotransferase (ALT), and aspartate aminotransferase (AST) were purchased from Nanjing Jiancheng (Nanjing Jiancheng Bioengineering Institute, Nanjing, China), and OD values were detected by an enzyme-labeled meter Mutiskan MK3 (Thermo Fisher Scientific, USA); mouse anti-*β*-actin antibody (66009) was purchased from Proteintech (Sanying Biotechnology, Wuhan, China), rabbit anti-PPAR*γ* antibody (GB112205) and HE staining kit (G1005) were purchased from Servicebio (Servicebio Biological Technology, Wuhan, China), and rabbit anti-ADRP antibody (DF7603) was purchased from Affinity Biosciences (Affinity Biosciences, Zhenjiang, China). Horseradish peroxidase-labeled goat anti-mouse secondary antibody (A21010) was purchased from Abbkine (Abbkine Scientific, Wuhan, China), and horseradish peroxidase-labeled alpaca anti-rabbit secondary antibody (HA1031) was purchased from HuaAn (HuaAn Biotechnology, Hangzhou, China).

### 2.3. Main Method

#### 2.3.1. Preparation of ICAE

*I. cornuta* leaves were collected from the Herbal Garden of Rizhao Campus, Jining Medical University. The picking time was in September, 2020, and after washing, the leaves were shade dried and then oven dried at 50°C. Decoctions of *I. cornuta* were prepared by traditional decoction method, and the dry powder of ICAE was obtained by freeze-drying method. Preparation of ICAE was performed as follows: the dried leaves were powdered using an electric blender and then the powder was sieved through a fine mesh to obtain finely powdered samples. About 30 g of the fine powder was macerated in 500 mL of distilled water for 30 min, then decocted for 1 hour; after the first decocting, the drug-containing solution was removed and the residue was boiled again twice for 1 hour each time; the decoctions were then combined and subjected to negative pressure filtration, and about 150 mL clarified decoction was obtained. The decoction was initially concentrated into 30 mL and then further concentrated in the rotary evaporator. Finally, the concentrated extract was dried with a SJIA-10N vacuum freeze dryer (Shuangjia, Ningbo, China). When the water was sublimated completely, the dry extract was ground into the brown-yellow dry powder immediately. ICAE dry powder was collected and sealed in 1.5 mL, stored in a refrigerator at −20°C, and prepared before use.

#### 2.3.2. Preparation of High-Fat Feed

The high-fat diet used to induce fatty liver in mice was homemade in the laboratory. The aim of this study was to develop an obese model with simple hypertriglyceridemia and fatty liver. It is reported that dietary cholesterol has an important role in the progression of NAFLD to NASH by inducing inflammation and fibrosis in liver. Thus, obesity, insulin resistance, and glucose intolerance are less pronounced compared to mice fed a similar diet without cholesterol. Therefore, we added a modest amount of cholesterol (0.2%) in the diet [25–27]. The HFD formula in the present study is similar to the traditional formula of our laboratory to induce obesity [14], and the induction time is extended to 10 weeks. The HFD formula is as follows: basic feed 66.8%, lard 20%, cholesterol 0.2%, egg yolk powder 10%, and starch 3%. Material preparation is as follows: the basic feed is crushed and sifted to get fine basal feed powder; yolks were taken out from the boiled egg, then dried, and crushed, to get fine yolk powder; lard was refined from fresh fat; tapioca starch was used to make a starch paste. The process is described as follows: firstly, the basal feed powder is mixed with yolk powder in a container and the cholesterol is dissolved in the heated lard. Then, the lard is added to the powder and grasped evenly. Finally, the starch paste is added and dough is made, cylindrical-shaped dough is formed by using the 5 mL syringe with the front end removed, and then cylindrical wet feed was dried at 50°C in a blast drying oven. In order to prevent deterioration, the feed to be dried and already dried was packed in sealable plastic bags and stored in a refrigerator at −20°C.

#### 2.3.3. Serum and Hepatic Biochemical Assays

Each blood sample of mice was given two hours for clotting at room temperature and then centrifuged at 3000 rpm for 20 min at 4°C to separate the serum. The serum was collected for the analysis of blood glucose, TG, TC, ALT, and AST by the corresponding commercial kit. Small pieces of frozen liver tissues were homogenized by using a hand-held micro electric homogenizer in 9-fold anhydrous ethanol on ice and centrifuged to separate the supernatant. The supernatants were collected and used for the measurements of hepatic TG and TC by commercial kits.

#### 2.3.4. Hematoxylin and Eosin Staining

According to the standard procedure, small pieces of hepatic tissue and epididymal adipose tissue were fixed in 4% paraformaldehyde for histopathological examination. Then, tissue samples were embedded into wax blocks after dehydration, clearing, and paraffin immersion. The section was set as 8 *μ*m thick and dyed according to the staining procedure in the HE staining kit. After dying, the sections were dehydrated with gradient ethanol and cleared with xylene, and microscopic observation was finally performed after sealed with neutral gum.

#### 2.3.5. Oil Red O Staining

Oil red O staining was performed according to standard protocols. After fixation, the samples of liver tissue were routinely dehydrated and embedded in an OCT medium (Sakura, USA), and 9 *μ*m cryosections were prepared using a Leica CM1950 cryostat. The tissue sections were affixed to the adhesive slides, dried properly, soaked in the oil red dye for 30 min, protected from light, and kept at room temperature. After washing and mounting, the sections were finally observed with a microscope.

#### 2.3.6. Immunoblotting

Liver tissue samples were lysed in sample buffer containing 62 mmol/L Tris-HCl, pH 6.8, 2% SDS, 0.1 mmol/L sodium orthovanadate, and 50 mmol/L sodium fluoride. The protein content was determined by the BCA assay. Equal amounts of proteins (30 *μ*g) were loaded and separated by 10% SDS polyacrylamide gel electrophoresis. The proteins were transferred to the PVDF membrane (Millipore, USA) by the wet transfer method, and then the membrane was sealed with 5% milk dissolved in TBST (0.05% Tween-20) at room temperature for 1 hour. The membrane was then incubated with PPAR*γ* (1:1000) or ADRP (1:2000) overnight at 4°C, after washing three times, the membrane was incubated with a secondary antibody, and after washing another three times, the membrane was detected by using enhanced chemiluminescence detection reagents (NCM Biotech, Suzhou, China). Anti-*β*-actin (1:10000) served as a loading control. The immunoblots were imaged using the iBright FL1000 Imaging System (Thermo Fisher Scientific, USA). The optical density and area of the protein band were calculated using NIH ImageJ software from band densitometry, and the density values were expressed as tested proteins/*β*-actin ratio for each sample.

### 2.4. Statistics Analysis

Data were expressed as mean ± SEM and statistically analyzed using the Prism 8.02 program. The results were analyzed by one-way analysis of variance followed by Tukey's honestly significant difference tests for multiple comparisons. *P* < 0.05 was considered statistically significant.

## 3. Results

### 3.1. ICAE Ameliorated HFD-Induced Obesity but Had No Influence on Food Intake

**Figure 1:**
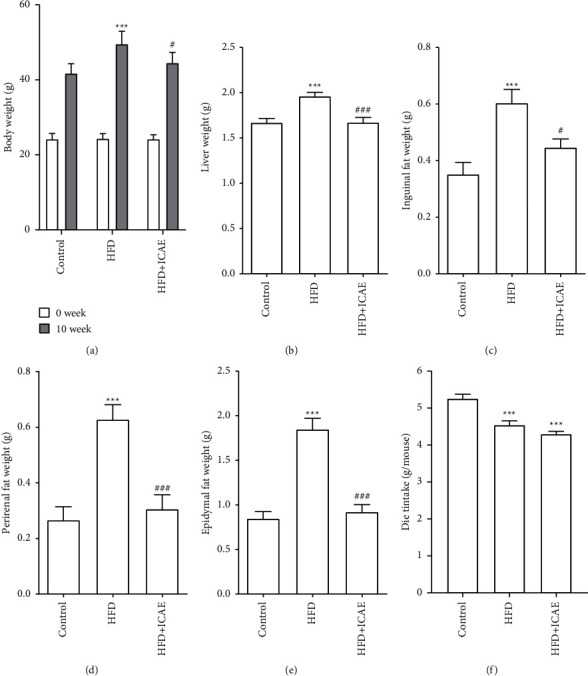
Effects of ICAE on body weight (a), liver weight (b), inguinal fat weight (c), perirenal fat weight (d), epididymal fat weight (e), and average food intake (f). Data are presented as mean ± SEM, *n* = 8–9. ^*∗∗∗*^*P* < 0.001*vs.* control group; ^#^*P* < 0.05, ^###^*P* < 0.001*vs.* HFD group.

Effects of ICAE on obesity and food intake were shown in Figure 1. About 10-week HFD feeding induced a significant increase in body weight, liver weight, and related fat accumulation in inguinal, epididymal, and perirenal fat tissues compared with mice of control group (*P* < 0.001), and ICAE treatment significantly reduced the above five indicators (*P* < 0.05, *P* < 0.001). In terms of food intake, the average 24 h food consumption of the HFD group and ICAE treatment group was lower than that of the control group (*P* < 0.001), but there was no significant difference between the two groups (*P* > 0.05).

### 3.2. ICAE Decreased the Elevation of Serum TG, TC, and Blood Glucose in HFD-Induced Mice

**Figure 2:**
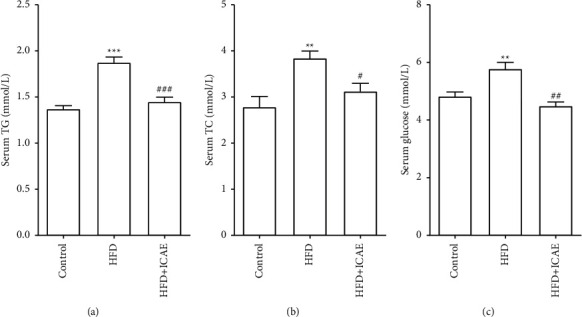
Effects of ICAE on serum TG (a), serum TC (b), and serum glucose (c) in HDF-induced mice. Data are presented as mean ± SEM, *n* = 8–9. ^*∗∗*^*P* < 0.01, ^*∗∗∗*^*P* < 0.001*vs.* control group; ^#^*P* < 0.05, ^##^*P* < 0.01, ^###^*P* < 0.001*vs.* HFD group.

Disturbance of lipid and glucose metabolism is a part of notable characteristics of obesity and fatty liver. As shown in Figure 2, 10 weeks of HFD induction led to a prominent increase in serum TC, TG, and glucose levels in the HFD group (*P* < 0.01, *P* < 0.001), while ICAE treatment significantly reversed the increase of serum TG, TC, and blood glucose levels induced by HFD (*P* < 0.05, *P* < 0.01, *P* < 0.001).

### 3.3. ICAE Reduced the Elevation of the Serum ALT and AST in HFD-Induced Mice

**Figure 3:**
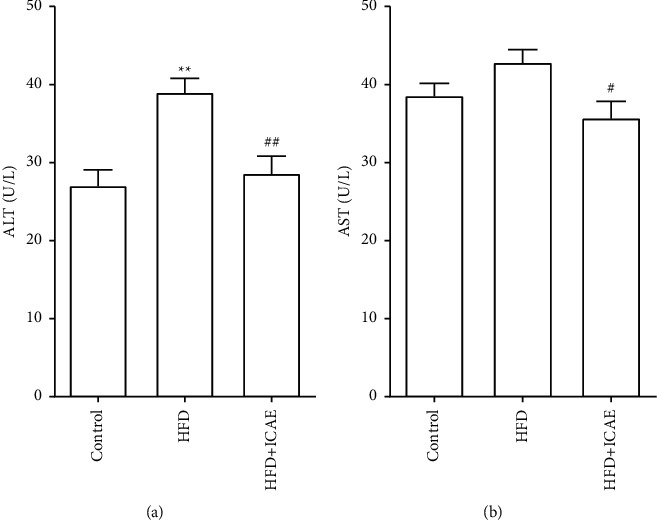
Effects of ICAE on serum ALT (a) and serum AST (b) on HDF-induced mice. Data are presented as mean ± SEM, *n* = 8–9. ^*∗∗*^*P* < 0.01*vs* control group; ^#^*P* < 0.05, ^##^*P* < 0.01, *vs.* HFD group.

Serum levels of alanine aminotransferase (ALT) and aspartate aminotransferase (AST) were determined as serum markers of hepatocyte damage. As demonstrated in Figure 3, serum ALT significantly increased in HFD mice (*P* < 0.01), and ICAE treatment dramatically reversed the elevation of ALT (*P* < 0.01). By contrast, the changes in AST were smaller than that in ALT. Serum AST of HFD group increased slightly compared with that in the control group, but there was no significant difference (*P* > 0.05). However, the AST level in the ICAE treatment group is lower than that in the HFD group (*P* < 0.05).

### 3.4. ICAE Attenuated Adipose Tissue Hypertrophy and Hepatic Steatosis in HFD-Induced Mice

**Figure 4:**
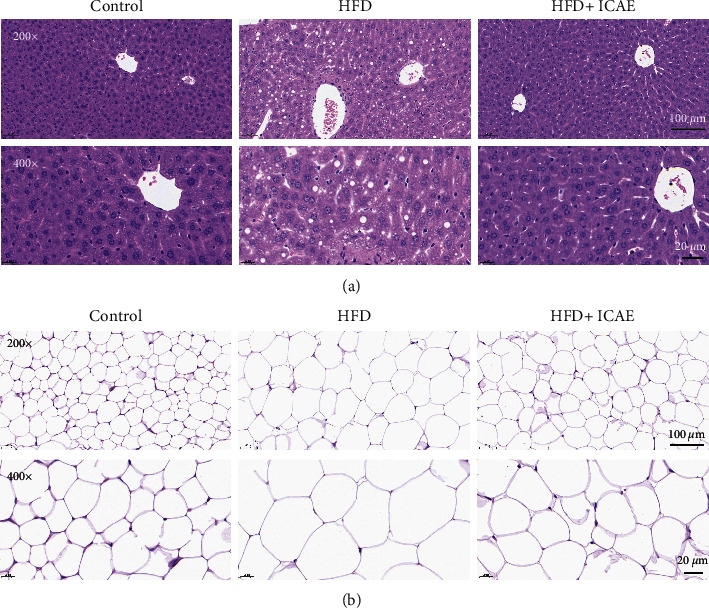
Influences of ICAE on liver and epididymal adipose tissue in HFD-induced mice. Photomicrographs of liver (a) and epididymal adipose tissue (b) of different groups with HE staining. Images were representative of three mice of diverse groups, which were taken at the magnification of 200 × (scale bar, 100 *μ*m) and 400 × (scale bar, 20 *μ*m), respectively.

The influences of ICAE on the morphology of adipose tissue and liver tissue were observed by HE staining (Figure 4). HE-stained sections of liver showed that hepatocytes of mice in the control group have very small vacuoles in the cytoplasm, while hepatocytes of mice in the HFD group have a large number of vacuoles, especially in the central venous area; ICAE treatment significantly decreased the number and diameter of vacuoles in hepatocytes. HE-stained sections of epididymal fat tissue showed that adipocytes in normal control mice were small in diameter and adipocytes of HFD mice expanded significantly, while ICAE treatment reversed the expansion of adipocytes.

### 3.5. ICAE Decreased Hepatic Lipid Accumulation in HFD-Induced Mice

**Figure 5:**
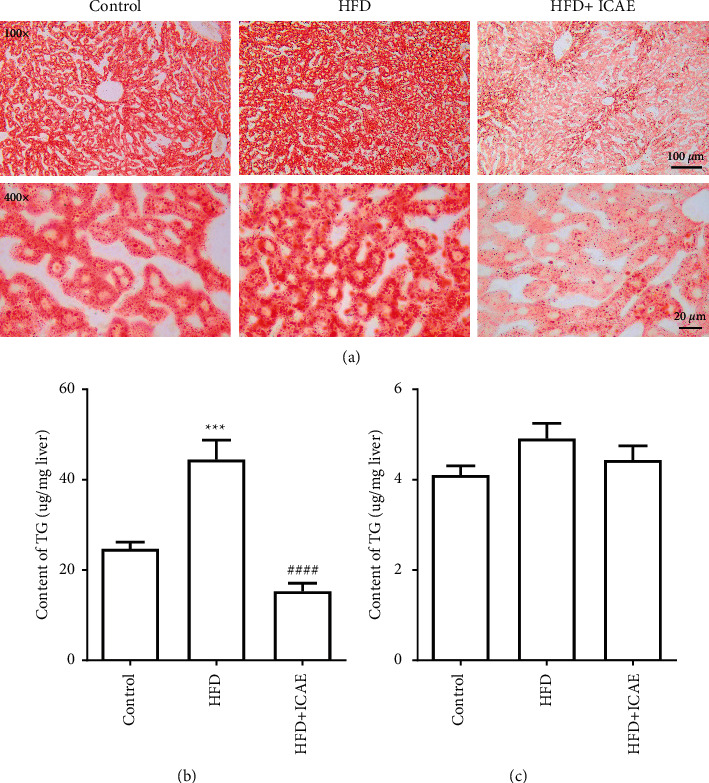
Effects of ICAE on lipid accumulation in liver of HFD-induced mice. (a) The lipid accumulation in liver of different groups was investigated by oil red O staining. Images were representative of three mice of diverse groups, which were taken at the magnification of 100 × (scale bar, 100 *μ*m) and 400 × (scale bar, 20 *μ*m), respectively. Effects of ICAE on the hepatic content of TG (b) and TC (c) of HFD-induced mice, *n* = 8–9. ^*∗∗∗*^*P* < 0.001 vs. control; ^####^*P* < 0.0001 vs. HFD group.

Hepatic TG and TC content assay and oil red O staining were used to evaluate the influences of ICAE on HFD-induced fatty liver. As shown in Figure 5, oil red O staining of livers showed that there were only a few of tiny lipid droplets in the hepatocytes of normal control mice and the lipid droplet numbers and sizes in liver tissue of HFD group were significantly increased, while HFD-induced steatohepatitis can be significantly reversed by ICAE treatment. The changes in hepatic content of TG and TC determined by the assay kit were consistent with the results from oil red O staining. The hepatic content of TG in HFD mice was 80% greater than that of control mice (*P* < 0.001), while the content of TG in the ICAE treatment group reduced to 34% of that in the HFD group (*P* < 0.0001). However, unlike TG, hepatic content of TC only slightly increased compared with the control group, and ICAE treatment showed some tendency to decrease the elevation, but there were no significant differences among the groups (*P* > 0.05).

### 3.6. ICAE Suppressed the Upregulation of PPAR*γ* and ADRP Expression in the Liver of HFD-Induced Mice

**Figure 6:**
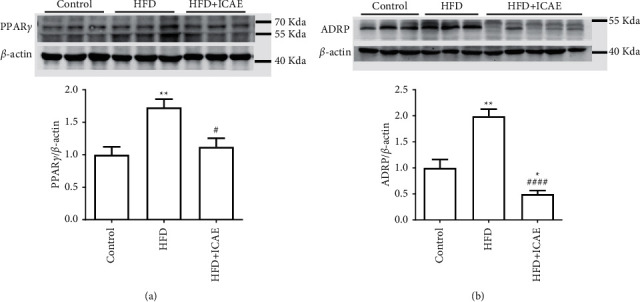
Effects of ICAE on the protein expression of PPAR*γ* and ADRP in the liver of HFD-induced mice. The protein levels of PPAR*γ* (a) and ADRP (b) were detected by immunoblot. Images were representative of three independent experiments, and average protein levels were quantified as ratios to *β*-actin, *n* = 5–6. ^*∗*^*P* < 0.05, ^*∗∗*^*P* < 0.01*vs* control; ^#^*P* < 0.05, ^####^*P* < 0.0001*vs.* HFD group.

To study the underlying mechanism of ICAE on fatty liver, we further observed the modulation of ICAE on the protein expression of PPAR*γ* and ADRP in liver. As shown in Figure 6, the protein level of PPAR*γ* was significantly increased by HFD induction (*P* < 0.01), and ICAE treatment obviously suppressed the upregulation of PPAR*γ* (*P* < 0.05). Furthermore, ADRP expression was also increased in the HFD group compared with the control group (*P* < 0.01), and similarly, ICAE treatment dramatically reduced the protein expression of ADRP (*P* < 0.0001). Moreover, it is worth noting that the protein expression of ADRP in the ICAE treatment group was strongly repressed, which was even lower than that in the control group (*P* < 0.05).

## 4. Discussion

### 4.1. ICAE Treatment Prevents the Fatty Liver in HFD-Induced Mice

NAFLD is considered to be the manifestation of metabolic syndrome in the liver and is related to metabolic risk factors such as obesity, dyslipidemia, hypertension, and diabetes. Therefore, an HFD-induced obese hepatic steatosis mouse model is an ideal fatty liver model, which can simulate the pathogenesis and mechanism of human fatty liver. In this experiment, 10-week HFD feeding induced a significant increase in body weight, fat mass, liver weight, serum TG, TC, and glucose, compared with mice of control group, and ICAE treatment significantly reduced all the indicators above. The results demonstrated that ICAE has brilliant antiobesity, hypoglycemic, and hypolipidemic effects, which were in agreement with the previous reports [13, 14].

Food intake is an important factor affecting weight changes. The present study showed that the average food intake of mice in the ICAE treatment group was slightly lower than that of the HFD group, but there was no significant difference. The results indicated that ICAE had no significant effect on food intake. The food intake of the mice in the HFD group and ICAE group was significantly lower than that in the control group. This may be due to the fact that feed rich in fat, cholesterol, and protein is more likely to make the mice feel full and prolonged gastric emptying.

More importantly, our findings illustrated that ICAE treatment significantly attenuated hepatic lipid deposition, especially TG content via multiple methods, including enzymatic determination of the hepatic lipid content, HE staining, and oil red O staining. Nevertheless, few changes were observed in hepatic TC content. Since cholesterol stored in the liver mainly comes from de novo synthesis and reflects the biosynthetic function of the liver, our results indicated that ICAE treatment specially reduced the hepatic TG content and had no influence on the hepatic biosynthesis of cholesterol.

Taken together, our findings demonstrated that ICAE treatment significantly reduced HFD-induced obesity, attenuated the elevation of serum TC, TG, and glucose, as well as lipid deposition in the liver, but have no influence on food intake. To our knowledge, we are the first to verify the preventive effects of ICAE on fatty liver in the HFD-induced obese hepatic steatosis mouse model.

### 4.2. ICAE Exerted Beneficial Effects upon Hepatic Injury Induced by HFD

Elevations in serum ALT and AST generally reflect hepatocyte injury. Elevated ALT level and a low AST/ALT ratio suggest nonalcoholic fatty liver disease and nonalcoholic steatohepatitis, increasing the risk of liver cirrhosis and hepatocellular carcinoma [28, 29]. The present study showed that serum ALT elevated significantly in the mice of HFD group; however, the level of AST, only increased slightly, did not reach a statistical difference. The result is in line with the clinical observation of patients with fatty liver [29]. More importantly, our findings demonstrated that ICAE treatment significantly reduced the elevation of ALT and AST compared with HFD mice. Therefore, the results indicated that on the one hand, ICAE treatment (at the dose of 3 g per kilogram, each day for 10 weeks) had no damage to liver function; on the other hand, ICAE not only had antifatty liver effects but also exerted beneficial effects upon hepatic injury induced by HFD.

### 4.3. Preventive Effects of ICAE on Fatty Liver Are Concerned with the Downregulation of PPAR*γ* and ADRP Protein Expressions in Liver

Since the preventive effects of ICAE on fatty liver have been identified, the underlying protective mechanisms were further investigated. It is reported that the expression of PPAR*γ* is specifically upregulated in the liver of HFD-induced mice [16, 30], and suppression of PPAR*γ* expression reduces fatty liver induced by HFD [17, 31]. Furthermore, Samuel et al. recently reported that PPAR*γ* agonist, rosiglitazone, promotes fatty liver via direct activation of hepatic PPAR*γ* in adult-onset hepatocyte-specific PPAR*γ*-knockout mice [32]. Moreover, our previous study confirmed that ICAE downregulates the protein level of PPAR*γ* and inhibits adipocyte differentiation in vitro [14]. In addition, Feng et al. isolated some bioactive substances from *I. cornuta*, which can inhibit the gene transcription of PPAR*γ* [23]. Therefore, we proposed that the antifatty liver effect of ICAE is possibly associated with PPAR*γ*. The present study demonstrated that ICAE treatment significantly suppressed the upregulation of PPAR*γ* expression in the liver of HFD-induced mice.

ADRP, which belongs to the PAT (perilipin, adipophilin, and TIP47) protein family, is the predominant PAT protein that coats small lipid droplets in nonadipocytes [33]. It is reported that ADRP is a target gene of PPAR*γ* and is upregulated in liver steatosis in both humans and mice [19, 21, 34]. The exact role of ADRP on fatty liver has been identified in ADRP-deficient mice [20, 35]. ADRP-deficient mice with normal diet display a 60% reduction in hepatic TG compared with wild-type mice and are resistant to HFD-induced fatty liver [20]. Furthermore, knockdown of ADRP expression in the liver of leptin-deficient mice relieves hepatosteatosis and improves whole-body insulin resistance [35]. Therefore, ADRP is also considered to be a potential target for the prevention and treatment of NAFLD.

In the present study, we found that ICAE treatment dramatically reduced the protein expression of ADRP in the liver of HFD-induced mice (Figure 6(b)). The result can be interpreted as follows: firstly, ADRP is the target gene of PPAR*γ*, and the lower expression of ADRP could be associated with the downregulation of PPAR*γ*; secondly, ADRP belongs to lipid droplet-associated proteins, and the abundance of ADRP protein is directly proportional to the levels of intracellular lipid [19, 21, 34, 36]. It is also of interest to note that the protein level of ADRP in the ICAE treatment group is even lower than that in the control group, which was in accordance with the change trends in the results from oil red O staining, HE staining, and enzymatic TG content assay. Although we could not provide an explanation for why ADRP expression and hepatic lipid deposition were suppressed so strongly by ICAE treatment, we can only conclude that the protective effects of ICAE on fatty liver are concerned with the downregulation of PPAR*γ* and ADRP protein expression, the suppression of ADRP is at least partly related to the downregulation of PPAR*γ* expression, and other mechanisms might exist to account for the antifatty liver effects of ICAE.

It should be noted that this is only a preliminary study, and the protective mechanisms of ICAE on fatty liver should be further studied on both in vivo and in vitro experiments. Whether other mechanisms participate in the preventive effects of ICAE on fatty liver? Whether ADRP and PPAR*γ* are the main executors of ICAE modulation, and what is the precise mechanism by which ICAE affects the protein expression of PPAR*γ* and ADRP? Whether PPAR*α* and PPAR*δ* contribute to the preventive effects of ICAE on fatty liver? In the follow-up study, we will design more rigorous experiments to address these issues.

## 5. Conclusion

The present study provided the first experimental evidence for the application of ICAE in the prevention of fatty liver. The results identified that ICAE protects against HFD-induced obesity, elevated serum lipid, glucose, and ALT in mice and has brilliant antifatty liver and hepatocellular protective effects. More importantly, we revealed a mechanism that ICAE may alleviate liver steatosis by reducing the protein expression of ADRP and PPAR*γ*. Because of these effects, Chinese medicine *I. cornuta* is expected to be a promising therapeutic option for the management of NAFLD.

## Data Availability

Data used to support the findings of this study are included within the article.
